# The Dental Misinformation Epidemic: An Evaluation of Quality, Accuracy and Educational Impact in Youtube's Tooth Bleaching Content

**DOI:** 10.1111/eje.70114

**Published:** 2026-02-23

**Authors:** Letícia de Azevedo Lima Silva, Hugo Juan da Silva Rolim, Gabriella Rodrigues da Silva, Pedro Henrique Sette‐de‐Souza, Moan Jéfter Fernandes Costa

**Affiliations:** ^1^ Departamento de Odontologia Universidade de Pernambuco Arcoverde/Pernambuco Pernambuco Brazil; ^2^ Programa de Pós‐Graduação Em Odontologia Universidade de Pernambuco Recife/Pernambuco Pernambuco Brazil

**Keywords:** aesthetics, dental, education, health communication, tooth bleaching

## Abstract

**Introduction:**

The proliferation of inaccurate information about tooth bleaching on the internet poses a growing risk to oral health, promoting potentially harmful practices while undermining evidence‐based professional guidance.

**Objective:**

Evaluate the quality, reliability, and educational impact of tooth bleaching videos on YouTube, specifically examining disparities between viewer engagement and scientific rigour.

**Methodology:**

An analysis was conducted on videos selected from the most searched terms on Google Trends. The assessment used the modified DISCERN, Global Quality Score (GQS) for overall quality, standardized audiovisual criteria and engagement metrics, analysed by two independent researchers.

**Results:**

71.4% of videos demonstrated low reliability (scores ≤ 2/5), while only 4.7% achieved maximum quality (5/5 GQS). Non‐professional content garnered 3.5 × greater engagement than videos produced by health professionals despite 30.2% scoring minimally for reliability (1/5). Audiovisual production quality was prioritized over accuracy, with 100% of videos rated as having high/moderate technical standards despite frequent misinformation. Discussion: YouTube's. algorithms disproportionately amplify sensationalized, unverified content over evidence‐based professional guidance. This promote clinical risks like predominant promotion of unproven methods (e.g., charcoal, lemon) may lead to enamel damage and hypersensitivity.

**Conclusion:**

It's urgent a call to action for dental professionals to actively create and disseminate scientifically validated digital content. Healthcare platforms and regulatory bodies must collaborate to develop strategies that prioritize quality and accuracy in dental health communication.

## 1 Introduction

Tooth bleaching is one of the most popular procedures in dentistry, capable of modifying tooth coloration and providing smile harmony [1]. This conservative aesthetic alternative requires no tooth reduction and presents lower costs compared to other procedures such as full crown fabrication or composite resin veneers [2]. Although demand for this procedure has grown in recent decades, it still raises relevant consumer concerns regarding dietary restrictions, postoperative pain, and cost. Consequently, three key questions persist in the public perception: (1) the white diet [3], (2) tooth sensitivity [4, 5] and (3) financial investment [6].

Parallel to this, recent years have witnessed an exponential rise in social media popularity as platforms for sharing ideas, knowledge, and experiences. Consequently, there has been growing demand for health‐related information on social media platforms [7]. In Brazil alone, approximately 60% of individuals aged 25–34 years reported searching for health information online in 2022 [8].

The emergence of online video platforms and audiovisual formats has paved the way for dynamic and engaging content delivery, enabling innovative narrative approaches to information dissemination [9]. As a result, YouTube has become the leading video‐sharing website globally and consequently a major social media platform, with one billion hours of content watched daily. Within this environment, users can access information while simultaneously creating and sharing diverse content, serving as mediators on a platform where the most popular channels boast over 50 million subscribers [10].

Among all existing social media platforms today, YouTube stands out for its distinctive feature of allowing long‐form videos. This format enables content creators to dedicate more time to thoroughly present research, sources, justifications, and other detailed information [11]. However, a significant drawback of this platform lies in its open publishing system, where anyone can share content without verification processes. Consequently, the quality and accuracy of shared information often become questionable. When used indiscriminately, YouTube can thus become one of the primary vehicles for disseminating false and/or misleading information [10].

Given this context, there is growing interest in investigating online content, as the uncontrolled proliferation of information makes it increasingly difficult for users to identify reliable sources and guidance when needed [12]. Numerous studies have evaluated YouTube videos across various dental topics, including tooth avulsion, fluoride use, halitosis, harmful oral habits, and dental caries [7, 13, 14, 15]. However, these investigations have consistently found such content to be of low quality, limited usefulness, and generally unreliable for educating the lay public.

This study aimed to conduct a rigorous analysis of tooth bleaching‐related informational videos available on YouTube, systematically evaluating their technical quality, scientific accuracy, and informational reliability. Additionally, we sought to assess whether these videos promote or hinder proper public understanding of tooth bleaching procedures by examining both their beneficial and detrimental aspects. The findings intend to contribute to the improvement of evidence‐based oral health education and information dissemination in digital media.

## 2 Methods

### 2.1 Study Design

An observational infodemiology study was conducted to analyse educational videos about tooth bleaching on the YouTube platform.

### 2.2 Search Strategy and Video Selection

The search terms were selected through filtering on the Google Trends platform (https://trends.google.com.br/trends/), a website that shows the most searched terms in the recent past across Google's system. The search term ‘tooth bleaching’ was entered into the site's search engine along with filters for location (Brazil), timeframe (last 12 months), and category (Health), applied specifically to the YouTube platform. This process identified the three most searched terms: ‘Clareamento dental’ [‘Tooth bleaching’], ‘Clarear dentes’ [‘Bleach teeth’] and ‘Creme dental clareador’ [‘Whitening toothpaste’]. Subsequently, the YouTube platform was used with the ‘Return YouTube Dislike’ extension to evaluate videos and extract data regarding the number of dislikes for each video.

This study prioritized Portuguese‐language YouTube content due to Brazil's unique dental ecosystem: the country hosts 8× more dental schools than the U.S. and 17× more than Canada [16] (Vieira & Castro, 2022) while research shows that 85.9% of its population desires tooth whitening [17] (Silva et al., 2018). Beyond that, Portuguese, the world's sixth most spoken language, digital health environments remain critically understudied. This research gap is particularly concerning given YouTube's algorithmically‐driven content bubbles, which propagate language‐specific misinformation unchecked by the dental community's substantial local presence. Our analysis addresses this disparity by systematically evaluating Portuguese bleaching content on the world's dominant video platform, YouTube.

The search on both Google Trends and YouTube was conducted in December 2024, using each term separately. Duplicate videos, those with content unrelated to the research objective, as well as videos longer than one hour or shorter than one minute were excluded. The duration limits were based on the premise that excessively long videos may compromise educational effectiveness for viewers, as they require greater attention and concentration—qualities that are often lacking in today's internet users due to the prevailing culture of immediacy and anxiety around content consumption. Conversely, it was also considered that very short videos, lasting less than 1 min, would be unable to provide detailed and complete information, making it difficult to meaningfully assess the quality, accuracy, and educational impact of such productions [18].

### 2.3 Data Collection

The videos were independently evaluated by two researchers (LALS and HJSR), with the data subsequently compiled in Excel spreadsheets. The assessment criteria included: (1) number of likes, (2) number of dislikes, (3) number of comments, (4) duration in seconds, (5) engagement rate calculated as (number of likes minus number of dislikes) divided by total views multiplied by 100%, (6) view rate determined by (number of views divided by number of days since video upload) multiplied by 100%, (7) video source categorized either as healthcare professionals (including dentists, dental students, and oral health technicians) or other sources (such as official institutions or digital influencers), (8) audiovisual quality rated as high, moderate, or low, (9) reliability scored on a 1–5 scale (with 1 being lowest and 5 highest), (10) usefulness scored 0–5 (with 0 being lowest and 5 highest), and (11) overall quality rated 1–5 (with 1 being lowest and 5 highest). This comprehensive evaluation framework allowed for systematic analysis across multiple dimensions of video content quality and performance.

The inter‐rater reliability analysis yielded excellent agreement between evaluators, with a Cohen's kappa coefficient of κ = 0.92 (95% CI: 0.89–0.94). This agreement level indicates highly consistent scoring between the independent assessors. The narrow confidence interval further reinforces the precision of this reliability estimate, suggesting minimal variability in the observers' interpretations of the assessment criteria. Such strong inter‐observer concordance supports the methodological rigour and reproducibility of the video evaluation process in this study.

### 2.4 Evaluation of Reliability, Usefulness, Overall Quality, and Audiovisual Features

The reliability of the videos was assessed using the modified DISCERN scale [19], which evaluates a material's ability to present consistent results while minimizing discrepancies with potential sources. This scale consists of five key criteria: (1) clarity of objectives, (2) use of reliable information sources, (3) impartiality, (4) inclusion of additional reference sources, and (5) acknowledgment of areas of uncertainty regarding the subject matter. Each criterion was scored dichotomously, with ‘yes’ responses receiving one point and ‘no’ responses zero points. The total reliability score for each video ranged from 0 to 5 points, with higher scores indicating greater reliability. This standardized assessment tool allowed for systematic evaluation of the informational quality across all video samples while maintaining methodological consistency with established protocols in health communication research.

Video usefulness was evaluated based on its practical value and informational content for viewers. A point was awarded for each accurately addressed key aspect: (1) differentiation between intrinsic and extrinsic tooth discoloration, (2) types of bleaching procedures, (3) typical symptoms (e.g., tooth sensitivity), (4) risks associated with home‐based methods, and (5) recommendation for professional dental supervision during bleaching treatments. This yielded a usefulness score ranging from 0 (minimal utility) to 5 (optimal utility), with higher scores indicating more comprehensive and clinically relevant information for potential patients.

The overall quality of the videos was assessed using the Global Quality Score (GQS), a standardized 5‐point scale where 1 represents poor quality content that is not useful for patients, 2 indicates generally low quality with poor flow and limited patient applicability, 3 corresponds to moderate quality with suboptimal flow and marginal usefulness, 4 denotes good quality with proper flow that is beneficial for patients, and 5 signifies excellent quality material that is highly useful for patients. This scoring system specifically evaluates both how effectively the information is presented and the continuity/flow of the video content, as documented in previous literature. The GQS provides a validated framework for assessing the educational value and communication effectiveness of health‐related video materials [20, 21].

The audiovisual quality [22] of the videos was categorized as good, moderate, or poor based on specific technical criteria. Videos demonstrating professional production standards—including clear visual aids, on‐screen text, professional graphics, seamless editing effects, and noise‐free audio with perfect comprehensibility—were classified as ‘good’. Home‐produced videos showing environmental interference (such as background traffic noise) or partially unclear narration were categorized as ‘moderate’. Videos exhibiting blurred or pixelated images, substantially incomprehensible content, or complete absence of audio were rated as ‘poor’. This tripartite classification system allowed for systematic evaluation of production values and their potential impact on information delivery and viewer comprehension.

### 2.5 Data Analysis

The statistical analysis was performed using Jamovi software version 2.3.28 (The Jamovi Project, Sydney, New South Wales, Australia). Descriptive analyses included calculation of absolute and relative frequencies, along with measures of central tendency (mean, median, mode) and dispersion (standard deviation). Frequency tables and bar charts were subsequently generated to present the study findings. All analytical procedures followed standardized protocols for infodemiological research to ensure methodological consistency and reproducibility of results. The visual representations were designed to optimize data interpretation while maintaining scientific accuracy in conveying the YouTube content evaluation outcomes.

### 2.6 Ethical Considerations

Since this study exclusively analysed publicly available information without involving human participants or private data, submission to an Institutional Review Board (IRB) for human subjects research was not required. The research complied with all applicable ethical guidelines for infodemiological studies utilizing publicly accessible digital content. All data were anonymized during analysis, and no attempts were made to identify or interact with video creators or viewers. This approach aligns with current international standards for ethical digital health research involving secondary analysis of open‐access online materials.

## 3 Results

### 3.1 Video Selection and Characteristics

The initial search yielded 366 videos, from which the following were excluded: videos exceeding 60 min (*n* = 3), those under one minute (*n* = 284), and off‐topic content (*n* = 36). Consequently, 43 videos were included in the analysis, distributed across the three search terms: 15 on ‘types of tooth bleaching’, 15 on ‘whitening toothpaste’, and 13 on ‘how to bleach teeth’.

The average video duration was 413.20 s (approximately 7 min 28 s), with mean values of 1273 likes and 318.1 comments per video. Notably, 44.2% of videos demonstrated low overall quality (scoring ≤ 2 on the GQS scale), and 84.21% of these low‐quality videos were published by non‐professional sources.

### 3.2 Engagement Patterns Across Bleaching‐Related Video Categories

Quantitative analysis of viewer engagement metrics revealed significant variation across video categories (Table 1). The ‘Bleaching teeth’ search term demonstrated markedly higher mean likes (3241 ± 7463) compared to ‘Types of Bleaching’ (137 ± 208) and ‘Bleaching Toothpaste’ (441 ± 495), though with substantial standard deviations indicating wide data dispersion. Dislike metrics followed a similar pattern, with ‘Bleaching teeth’ videos accumulating significantly more dislikes (557 ± 974) than other categories. Notably, ‘Bleaching Toothpaste’ videos generated the highest mean comments (604 ± 1995), suggesting particular viewer interest in this product category. The considerable standard deviations across all metrics highlight the heterogeneous nature of viewer engagement with dental bleaching content on digital platforms.

**TABLE 1:** Engagement metrics by video category.

Search Term	Videos (*n*)	Likes (mean ± SD)	Dislikes (mean ± SD)	Comments (mean ± SD)
Types of Bleaching	15	137 ± 208	10.0 ± 25.1	33.3 ± 80.7
Bleaching Toothpaste	15	441 ± 495	277 ± 948	604 ± 1995
Bleaching teeth	13	3241 ± 7463	557 ± 974	317 ± 287

*Note:* Self‐authored.

Abbreviation: SD, standard‐deviation.

### 3.3 Analysis of Video Quality by Content Category

The quality assessment of analysed videos revealed significant disparities across content categories (Table 2). Videos retrieved using the search term ‘Bleaching Teeth’ demonstrated the poorest quality outcomes, with 30.2% (*n* = 13) scoring the lowest possible quality rating (score = 1). In contrast, only content categorized under ‘Types of Bleaching’ achieved the highest quality scores (11.6%, *n* = 5, score = 5). Notably, ‘Bleaching Toothpaste’ videos showed moderate quality distribution, with the majority (16.3%, *n* = 7) receiving intermediate scores (score = 3). The cumulative percentage analysis indicates that 44.2% of all videos scored ≤ 2 on the quality scale, suggesting substantial room for improvement in overall content quality.

**TABLE 2:** Frequency of the general quality of the videos analysed.

Quality score	Videos analysed	Count (n)	% of Total
1 (lowest)	Bleaching Toothpaste	3	7.0%
	Bleaching Teeth	13	30.2%
	Types of Bleaching	0	0.0%
2	Bleaching Toothpaste	1	2.3%
	Bleaching Teeth	0	0.0%
	Types of Bleaching	2	4.7%
3	Bleaching Toothpaste	7	16.3%
	Bleaching Teeth	0	0.0%
	Types of Bleaching	4	9.3%
4	Bleaching Toothpaste	4	9.3%
	Bleaching Teeth	0	0.0%
	Types of Bleaching	4	9.3%
5 (Highest)	Bleaching Toothpaste	0	0.0%
	Bleaching Teeth	0	0.0%
	Types of Bleaching	5	11.6%

*Note:* Self‐authored.

### 3.4 Reliability Assessment of Dental Bleaching Content

The reliability analysis revealed significant disparities across video categories (Table 3). Content retrieved using the search term ‘Bleaching Teeth’ demonstrated particularly concerning results, with 30.2% (*n* = 13) scoring only 2 on the reliability scale and none achieving scores ≥ 4. In contrast, ‘Types of Bleaching’ videos showed moderate reliability (30.2%, *n* = 13 scoring 3). Notably, only ‘Bleaching Toothpaste’ content reached the highest reliability level (4.7%, *n* = 2, score = 5). The cumulative analysis indicates that 44.2% of all videos scored ≤ 2, reflecting substantial reliability limitations in available content.

**TABLE 3:** Frequency of reliability of analysed videos.

Reliability score	Videos analysed	Counts (*n*)	Percentage (%)
1 (Unreliable)	Types of Bleaching	0	0.0%
	Bleaching Toothpaste	4	9.3%
	Bleaching Teeth	0	0.0%
2	Types of Bleaching	0	0.0%
	Bleaching Toothpaste	2	4.7%
	Bleaching Teeth	13	30.2%
3	Types of Bleaching	13	30.2%
	Bleaching Toothpaste	5	11.6%
	Bleaching Teeth	0	0.0%
4	Types of Bleaching	2	4.7%
	Bleaching Toothpaste	2	4.7%
	Bleaching Teeth	0	0.0%
5 (Most reliable)	Types of Bleaching	0	0.0%
	Bleaching Toothpaste	2	4.7%
	Bleaching Teeth	0	0.0%

*Note:* Self‐authored.

### 3.5 Usefulness Assessment of Dental Bleaching Videos

Overall usefulness outcomes of the analysed videos are presented in Table 4. A considerable proportion of the content demonstrated low educational value, particularly among videos retrieved using the keywords ‘Bleaching Teeth’ and ‘Bleaching Toothpaste’, where 13 videos (30.2%) and 5 videos (11.6%), respectively, received the lowest usefulness score (0). In contrast, videos associated with the keyword Types of Bleaching more frequently achieved moderate usefulness ratings, with scores of 2 observed in 5 videos (11.6%) and scores of 5 also in 5 videos (11.6%). Intermediate usefulness scores were less frequent overall, with scores of 3 observed in 2 videos (4.7%) for Types of Bleaching and 3 videos (7.0%) for Bleaching Toothpaste. These results indicate that users searching for tooth bleaching information are often exposed to videos with limited educational usefulness, highlighting important gaps in the quality of online dental health information.

**TABLE 4:** Usefulness of the videos analysed, according to the keywords.

Usefulness Score	Content category	Count (*n*)	Percentage (%)
0	Types of Bleaching	0	0.0%
	Bleaching Toothpaste	5	11.6%
	Bleaching Teeth	13	30.2%
1	Types of Bleaching	1	2.3%
	Bleaching Toothpaste	0	0.0%
	Bleaching Teeth	0	0.0%
2	Types of Bleaching	5	11.6%
	Bleaching Toothpaste	7	16.3%
	Bleaching Teeth	0	0.0%
3	Types of Bleaching	2	4.7%
	Bleaching Toothpaste	3	7.0%
	Bleaching Teeth	0	0.0%
4	Types of Bleaching	2	4.7%
	Bleaching Toothpaste	0	0.0%
	Bleaching Teeth	0	0.0%
5	Types of Bleaching	5	11.6%
	Bleaching Toothpaste	0	0.0%
	Bleaching Teeth	0	0.0%

*Note:* Self‐authored.

### 3.6 Association Between Audiovisual Quality and Viewer Engagement

As shown in Figure 1, videos classified as having high audiovisual quality achieved substantially greater engagement compared with those rated as moderate quality, regardless of content source. Among videos produced by non‐health professionals, high audiovisual quality content reached an average engagement of approximately 4500 interactions, whereas moderate‐quality videos achieved around 1000 interactions. In contrast, videos produced by health professionals showed considerably lower engagement, with high‐quality productions reaching approximately 1000 interactions and moderate‐quality videos averaging close to 100 interactions. These findings demonstrate that engagement was consistently higher for content produced by non‐professionals and was strongly associated with audiovisual quality rather than source credibility.

**FIGURE 1:**
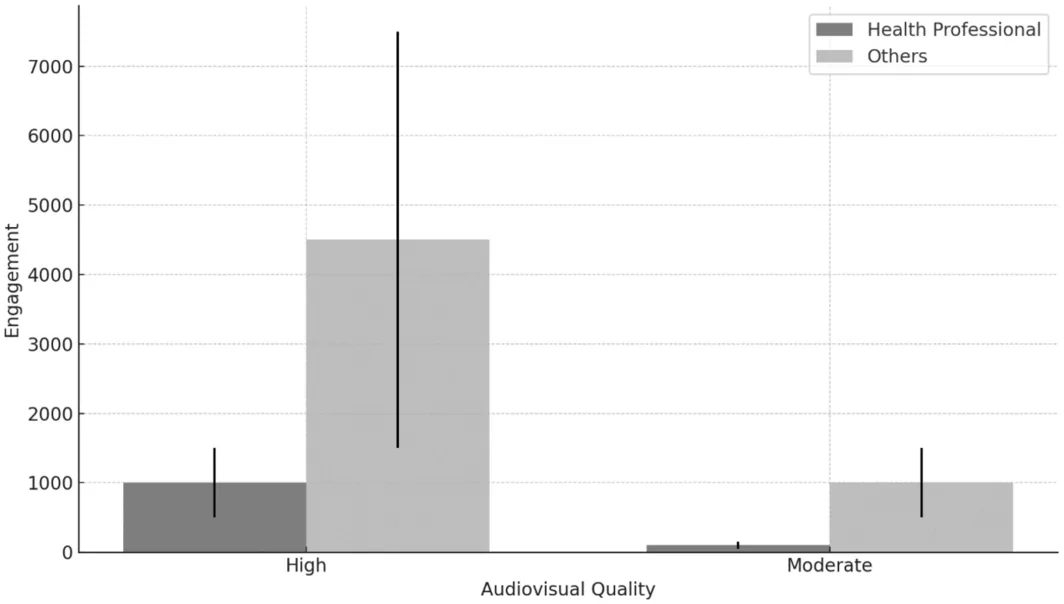
Audiovisual quality of the videos analysed, according to source and engagement. Self‐authored.

## 4 Discussion

This comprehensive analysis of dental bleaching content on YouTube reveals a troubling disconnect between scientific validity and popular engagement. The findings demonstrate that: (1) 71.4% of videos scored ≤ 2 in reliability and usefulness assessments, (2) ‘Bleaching Teeth’ searches yielded the least reliable content (30.2% scoring 1/5), while (3) only 4.7% achieved maximum quality scores (5/5). Paradoxically, videos from healthcare professionals—though technically superior—showed 3.5 × lower engagement than influencer‐generated content. These results expose systemic deficiencies in dental health communication, where algorithmically amplified misinformation outperforms evidence‐based guidance in both reach and audience interaction.

The videos retrieved using the search term ‘Bleaching Toothpaste’ demonstrated the highest average number of comments, underscoring how commercial brands strategically leverage digital influencers' popularity to introduce new products in this category, establish promotional partnerships, and enhance brand recognition among consumers [23] However, it is essential to emphasise that the actual therapeutic efficacy of toothpaste primarily depends on its capacity to release fluoride during brushing [24]. A comprehensive analysis of 50 charcoal‐based bleaching toothpastes available on Google.com and Amazon.com revealed that merely 8% contained fluoride in their formulations [25]. This significant discrepancy between marketed claims and clinical effectiveness raises important concerns about consumer misinformation and potential oral health risks associated with these widely promoted products.

Extensive research has been conducted to assess the effects of activated charcoal‐containing toothpastes on dental hard tissues. Current evidence indicates that while these products may produce subjective whitening effects that satisfy certain consumers, their mechanism of action is fundamentally abrasive in nature. This abrasiveness results in several detrimental outcomes: irreversible loss of tooth structure, increased surface roughness, reduction in enamel microhardness, and when dentine becomes exposed, significant hypersensitivity [26, 27, 28, 29]. These findings strongly suggest that charcoal‐based dentifrices, despite their commercial popularity, may pose unacceptable risks to long‐term oral health when used as part of routine hygiene practices. Dental professionals should be aware of these risks when advising patients seeking cosmetic whitening solutions.

Google Trends data from 2023 identified significant public interest in searches for homemade bleaching methods, including ‘bleach teeth with baking soda’, ‘dental bleaching recipe’, and other non‐professional approaches [30]. This study's analysis of corresponding YouTube content revealed these videos predominantly promoted unverified mixtures of pharmaceutical and food products (particularly sodium bicarbonate and lemon), while making scientifically unsupported claims about caries prevention and periodontal benefits [31]. Despite receiving high engagement metrics, all such content scored minimally (0–1) in reliability and usefulness assessments, exemplifying how digital platforms amplify health misinformation with detrimental consequences [32]. This phenomenon parallels established patterns of medical misinformation dissemination, including anti‐vaccine movements and fluoride opposition [33], and specifically erodes trust in professional dental care by propagating the dangerous misconception that bleaching requires only commercial products rather than clinical expertise. The findings collectively demonstrate how algorithmic promotion of unsubstantiated ‘DIY’ dental advice simultaneously jeopardizes oral health outcomes and undermines the professional standing of dental practitioners.

The analysis revealed a critical dissonance between content quality and public engagement: only 4.7% (*n* = 2) of videos achieved maximum reliability scores and 11.6% (*n* = 5) demonstrated excellent overall quality, yet these evidence‐based productions showed significantly lower engagement than their unreliable counterparts [34, 35, 36]. This pattern establishes a self‐reinforcing misinformation ecosystem where algorithmically amplified, low‐quality content promoting unsupervised bleaching protocols e.g., unsubstantiated claims about pigmenting beverages [37] or light‐activated systems [38] dominates public discourse, while scientifically validated information about desensitizing protocols [39] and professional care remains marginalized. The professional community's inadequate digital engagement exacerbates this crisis, with 57.7% of dentist‐published videos failing to meet basic usefulness standards (scores ≤ 2), reflecting either knowledge gaps about current evidence [37, 38, 39] or willful non‐dissemination of available science. This dual failure—both technological and professional—has cultivated an environment where the platform's inherent pedagogical potential [15] remains unrealized, allowing pseudoscientific narratives to flourish through cyclical consumption patterns that prioritize algorithmic virality over clinical validity.

Regarding the Overall Quality criterion, the analysis revealed a high frequency of level 1 productions—characterized by poor quality and clinically useless information—totalling 16 videos (37.2% of the sample). This proportion significantly exceeds the number of videos classified as good or excellent (scores of 4–5), which comprised only 13 videos (30.2%). Notably, all evaluated videos demonstrated exclusively high or moderate audiovisual quality, with no productions exhibiting poor audio or visual characteristics. These findings align with previous studies [7] and underscore the prevailing overvaluation of production quality at the expense of evidence‐based content. The data suggest substantial investments in visually appealing elements that engage viewers, while failing to provide adequate instructional value or clinically validated information.

Some limitations should be acknowledged in this research, such as the fact that the analysis was restricted to Portuguese‐language videos, which may limit the generalizability of the results to other linguistic and cultural contexts. The evaluation was based on standardised scoring systems (e.g., DISCERN, GQS), which, although validated, may not capture all the nuances of audiovisual health communication. In addition, the cross‐sectional design provides only a snapshot of the content available and the dynamic nature of social media means that the results may not reflect current uploads. Finally, although the study identified patterns of engagement, it did not assess actual viewer understanding or behavioural outcomes, which would require complementary experimental or survey‐based research. These limitations highlight the opportunities for future studies to broaden the platform's linguistic and methodological scope.

The results of this study reveal a worrying landscape of information on tooth whitening on YouTube, characterised by a predominance of low‐quality, unreliable content that significantly outweighs evidence‐based productions. The resulting information ecosystem not only puts patient safety at risk through the promotion of potentially harmful practices, such as abrasive charcoal toothpastes (only 8% contain fluoride), but also contributes to the wider de‐professionalisation of dentistry. These results underline the urgent need for better professional engagement with digital platforms and stricter content moderation policies to prioritise evidence‐based dental education over sensationalist and viewer‐oriented content. Future interventions should address both content quality and public health literacy to bridge the gap between scientific knowledge and public understanding of tooth whitening procedures.

## 5 Conclusion

The analysis revealed that most videos exhibited deficient quality, reliability, and practical utility, highlighting the crucial need to incentivize dental professionals to disseminate evidence‐based content utilizing credible sources and theoretical frameworks. This strategic approach would strengthen YouTube's educational capacity, counteract misinformation proliferation, and discourage unsafe, ineffective home bleaching techniques.

## Funding

The authors have nothing to report.

## Conflicts of Interest

The authors declare no conflicts of interest.

## Data Availability

The data that support the findings of this study are available from the corresponding author upon reasonable request.
